# Supplementary material for: Comparison of Patients Classified as High-Risk between International Cardiovascular Disease Primary Prevention Guidelines
Source: J Clin Med. 2024 Jul 26;13(15):4379. doi: 10.3390/jcm13154379 (PMC11312975; doi:10.3390/jcm13154379)
Supplement: Supplementary file 1 [file jcm-13-04379-s001.zip › jcm-3102393-supplementary.pdf]

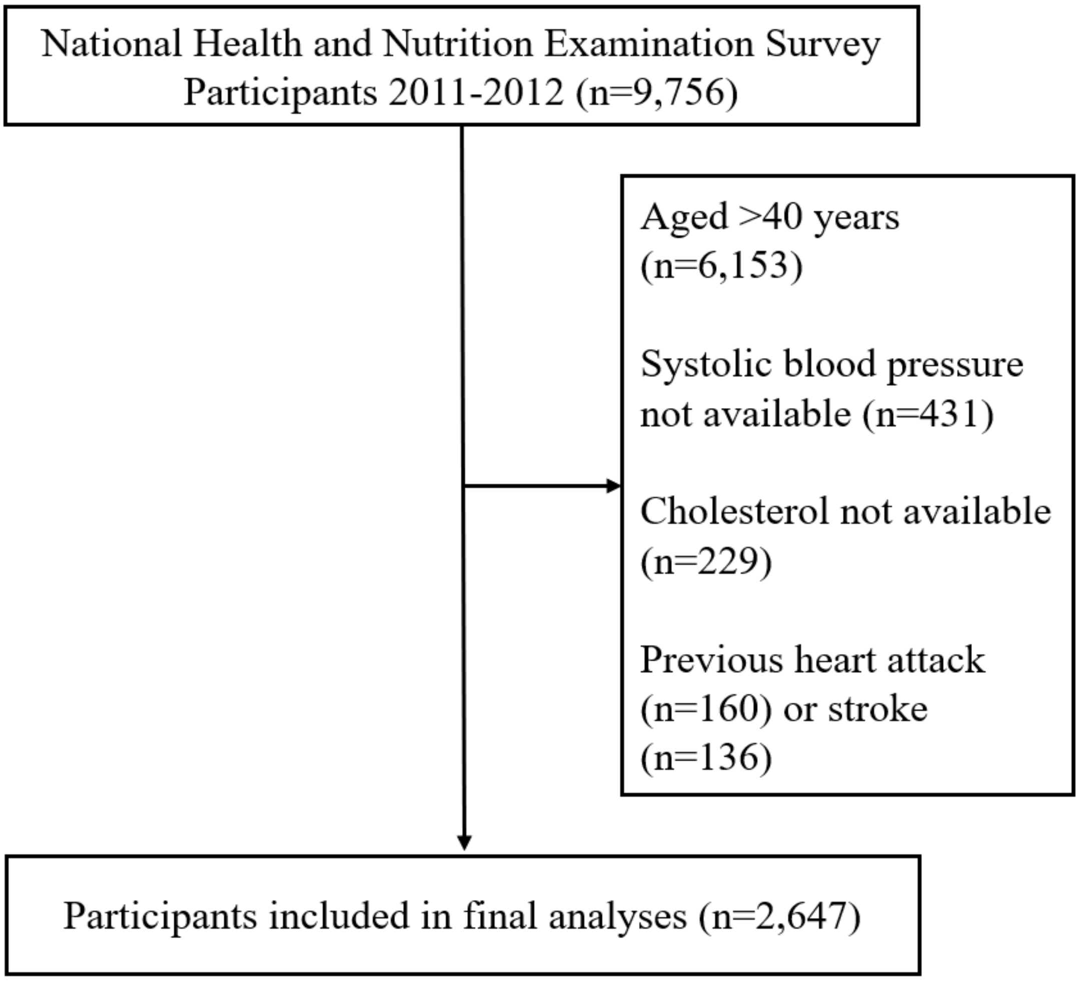

Figure S1. Participant flow chart.

Supplementary Table S1. Proportion of participants recommended for treatment according to cardiovascular disease primary prevention guidelines from Australia, England and the United States in adults aged over 40 years (n=2,647).

**Guideline recommendation**	**n (%)**
**AUSTRALIAN GUIDELINES**	
**High risk clinical criteria**	n=2,647
Diabetes aged ≥60 years	276 (10.4)
Diabetes and systolic blood pressure ≥140 mmHg	129 (4.9)
Blood pressure ≥180/110 mmHg	33 (1.3)
Chronic Kidney Disease*	105 (4.0)
Familial hypercholesterolaemia+	3 (0.1)
Total cholesterol ≥7.5 mmol/L	63 (2.4)
Total	455 (17.2)
**Absolute CVD risk estimation[x]**	n=2,192
5-year FRE >10-15% BP ≥140/90	109 (5.0)
5-year FRE >15% aged 45 years or older	141 (6.4)
Total	250 (11.4)
**Total recommended for treatment**	705 (26.6)
**ENGLISH GUIDELINES**	
**High risk clinical criteria**	n=2,647
Diabetes (type 1 or type 2)	446 (16.9)
Blood pressure ≥160/100 mmHg aged <80 years	116 (4.4)
Blood pressure ≥150/90 mmHg aged ≥80 years	58 (2.2)
Chronic Kidney Disease*	105 (4.0)
Familial hypercholesterolaemia+	3 (0.1)
Total	648 (24.5)
**Absolute CVD risk estimation[x]**	n=1,999
10-year QRISK2 >10% BP ≥140/90 mmHg	171 (8.6)
10-year QRISK2 >10%	628 (31.4)
Total	628 (31.4)
**Total recommended for treatment**	1,276 (48.2)
**UNITED STATES GUIDELINES**	
**High risk clinical criteria**	n=2,647
Diabetes aged 40-75 years	379 (14.3)
Diabetes and blood pressure ≥130/80 mmHg	247 (9.3)
Blood pressure ≥140/90 mmHg and absolute CVD risk <10%	221 (8.4)
Chronic kidney disease* and blood pressure ≥130/80 mmHg	53 (2.0)
LDL ≥190 mg/dl	36 (1.36)
Total	685 (25.9)
**Absolute CVD risk estimation[x]**	n=1,962
10-year PCE >10% and BP ≥130/80 mmHg	428 (21.8)
10-year PCE >7.5% and 1 or more risk enhancing factor**	321 (16.4)
10-year PCE ≥20%	352 (17.9)

Total	633 (32.3)
**Total recommended for treatment**	1,318 (49.8)

Abbreviations: LDL, low-density lipoprotein; FRE, Framingham Risk Equation; PCE, Pooled Cohort Equation.

*All reported cases of chronic kidney disease were used as it was not possible to distinguish whether or not cases were moderate or severe.

+Diagnosed familial hypercholesterolaemia was not available, total cholesterol ≥9.5mmol/L was used as a proxy indicator.

[x]Guidelines recommended absolute cardiovascular risk estimation is undertaken among those without clinical characteristics that indicate a recommendation for treatment without the need for absolute CVD risk estimation. As such, the proportion refers to those classified as high risk according to absolute risk estimation without high risk clinical characteristics and the denominator excludes those with these characteristics.

**Intermediate risk is recommended for treatment in the presence of risk enhancing factors that include family history of CVD; LDL-cholesterol 160-189 mg/dL; metabolic syndrome; chronic kidney disease; chronic inflammatory conditions including psoriasis and rheumatoid arthritis; and history of premature menopause, gestational diabetes or preeclampsia.
